# The Gateways of Medicines

**Published:** 1890-09-13

**Authors:** 


### The Gateways of Medicine.

October is the month when young men who intend to be doctors usually begin their professional studies. In the course of a year a good many letters are addressed to The Hospital and other medical papers by parents and youths asking for guidance. The medical papers respond to this desire for information by publishing annually in September a "Students' Number." The *British Medical Journal* and the *Lancet* give very full accounts of the different Universities and examining boards which confer degrees, and also of the various hospitals and medical schools at which students are instructed and prepared for examination. Space does not permit us to offer such detailed accounts; but in accordance with our usual custom, we give here a condensed statement which it is believed will answer all practical purposes.

There are many gateways by which the medical profession may be entered, but there are no back doors. The different qualifications which entitle men to be registered as medical practitioners to practise the art of medicine vary considerably in dignity and worth, but they are all respectable. The General Medical Council of Great Britain and Ireland is charged with the responsibility of the education and examination of all men who enter the profession, and the council, in spite of its anomalous constitution, discharges its duties with intelligence, fidelity, and zeal. It is certain that every duly qualified and registered medical practitioner who is a man of good private character is worthy of the confidence of the public.

### The Preliminary Examination.

Before a youth can properly commence medical study, he must be registered as a medical student, and in order to be so registered he must pass a preliminary examination in general education. There are several universities and examining boards at which the preliminary examination may be passed, and the examination varies in severity according to the medical degree which is finally to be taken. The first thing for a young man to do is to decide what degree he will take. He can then select the examining Board before which he will appear for his preliminary. If he be an English student he may propose to take the M.D. of Oxford, Cambridge, London, Victoria, or Durham; or if he be not so ambitious he will be content with the membership of the College of Surgeons, or to become a licentiate of the College of Physicians or of the Apothecaries' Society. When he has made up his mind what degree he will take, he should write to the dean of the medical faculty of the selected university, or to the secretary of the College of Physicians or Surgeons or of the Apothecaries' Society. By thus writing to head-quarters he will gain exactly the knowledge he wants without confusing himself with a variety of superfluous information.

If he be a Scotch student, or an Irish, or an Englishman who wishes to study in Scotland or Ireland, he will write to the dean of the medical faculty of the particular university whose degree he covets; or to the secretary of the College of Physicians or Surgeons which he selects. In every case he will receive at once the fullest information he may desire.

### Professional Education.

We will suppose the intending student to have passed his preliminary examination. What is the next thing to do? He must register himself as a medical student. There are three Registrars in the United Kingdom: one in London, for England; one in Edinburgh, for Scotland; and one in Dublin, for Ireland. In order to be registered, all that is to be done is to make application to one or other of those Registrars for the usual form, and to return it to him duly filled up and attested. It is quite necessary to be registered before beginning medical study; because studies commenced before registration do not count as part of the four or five years of compulsory study which the law exacts from every medical student prior to the taking of his qualification.

The student is now registered and competent to begin his work. What is the next step to be taken? He has selected the degree he will strive for. If it be a university degree, he will now go to the particular university whose degree he intends to take; and he will submit himself for the full period of his professional studies to the guidance of the university authorities. It should here be stated that the Universities of Oxford and Cambridge demand that their students shall take the B.A. degree at those Universities before or during their medical curriculum. In other words, no man can become a Bachelor or Doctor of Medicine of Oxford or Cambridge without first becoming a Bachelor or Master of Arts of the university whose medical degree he aspires to.

But the majority of English students do not enter for university degrees; and they study at hospitals which have recognised medical schools in connection with them. There are a great many such hospitals in London and the country. The following are the chief in England: Guy's, St. Bartholomew's, St. Thomas', the London, St. George's, University College, Charing Cross, King's College, Middlesex, St. Mary's, and Westminster, in London: and the Bristol Medical School, the Leeds School, Queen's College (Birmingham), Owen's College (Manchester), University College (Liverpool), the Newcastle Medical School, and the Sheffield School of Medicine, in the country. All these are recognised medical schools, and their lectures and classes qualify for the English Colleges of Surgeons and Physicians, the Scotch Colleges of Surgeons and Physicians, the Apothecaries' Society; and to a certain limited extent, for the Scotch universities.

The principal places of medical study in Scotland are the University of Edinburgh, the University of Glasgow, the University of Aberdeen, and the University of St. Andrew's, with the Extra-mural School of Edinburgh, and the Royal Infirmary Medical School, and St. Mugo's College, Glasgow. The Irish Medical Schools are the University of Dublin, the Royal University, and the Royal College of Physicians in Dublin.

The medical schools in each of the three countries vary greatly in reputation, and in the eminence and capacity of their teachers; and the student who has time and money at command will be well advised to seek one of the more famous schools of medicine in London, Edinburgh, or Dublin, for his scientific and practical work. But the young man whose chief ambition it is to be a useful general practitioner may safely consult his pocket in his choice of a university or hospital. Because all the medical schools of the three kingdoms are so well provided with teachers, that every student who works with conscience and intelligence can at any one of them lay the foundations of a competent medical education. Let no man therefore be afraid of entering the medical profession because he cannot pass through the university gates of Oxford or of Cambridge; of London or of Edinburgh. There are millions of people in this country who need plain and homely doctors, and who cannot pay those of any other kind. No young man need wish a more honourable or happy career than to minister to the poor in the time of sickness, and to receive their heartfelt gratitude and confidence. It is more than probable that when the final accounts of life are made up, many a doctor whose life has been spent among those who had little money to reward him with will take much higher honours and distinctions than some who have filled conspicuous places in the world. He who doubts this should not be a doctor.

Cost of Education.

The cost of obtaining a medical degree varies with the qualification sought for, and with the school at which the medical studies are pursued. A diligent and economical man may gain the licentiateship of the Apothecaries' Society in three and a-half years, and at an outlay of £350. He will hardly secure the M.B. of Oxford in less than seven years, or at a less cost than £1,000. The M.B. of London may be gained in five years, and the M.B. of Edinburgh in the same period; and the outlay for each of these latter degrees need not be more than from £600 to £700. The degrees of the other Scotch universities may be obtained at a still smaller cost—perhaps for about £450; and the diplomas of the English Colleges of Surgeons and Physicians at about the same amount, or a little less. The fees vary at the different London and provincial hospitals as well as at the universities. But in every case where money is a consideration the course to pursue is to write to several universities or hospitals and to ask for a prospectus of the medical curriculum of each. A comparison can then be made, and the particular school selected which suits the individual case.

Changes Made and Making.

It has hitherto been possible for the bulk of medical students to take their qualifying degree in four years from the date of registration. That is still possible; but it will be no longer so after January 1, 1892. The General Medical Council, which, as we have explained, is the controlling authority in medical education, has recommended a new regulation, and it will come into force at the date named. Practically, indeed, it will begin to operate at once; for all medical teachers are convinced of the difficulty and almost impossibility of endowing the average student with a sufficient knowledge of the general principles and facts of the various sciences on which the medical and surgical arts are founded in a shorter period than five years.

